# Prevalence and incidence of sarcopenia in the very old: findings from the Newcastle 85+ Study

**DOI:** 10.1002/jcsm.12157

**Published:** 2016-11-16

**Authors:** Richard Matthew Dodds, Antoneta Granic, Karen Davies, Thomas B. L. Kirkwood, Carol Jagger, Avan Aihie Sayer

**Affiliations:** ^1^ Academic Geriatric Medicine, Faculty of MedicineUniversity of SouthamptonSouthamptonSO16 6YDHampshireUK; ^2^ Ageing Geriatrics and Epidemiology, Institute of NeuroscienceNewcastle UniversityNewcastle upon TyneNE4 5PLTyne and WearUK; ^3^ NIHR Newcastle Biomedical Research CentreNewcastle University and Newcastle upon Tyne NHS Foundation TrustNewcastle upon TyneNE4 5PLTyne and WearUK; ^4^ Institute for AgeingNewcastle UniversityNewcastle upon TyneNE4 5PLTyne and WearUK; ^5^ Institute for Cell and Molecular BiosciencesNewcastle UniversityNewcastle upon TyneNE1 7RUTyne and WearUK; ^6^ Institute of Health and SocietyNewcastle UniversityNewcastle upon TyneTyne and WearUK; ^7^ MRC Lifecourse Epidemiology Unit, Faculty of MedicineUniversity of SouthamptonSouthamptonSO16 6YDHampshireUK; ^8^ NIHR Collaboration for Leadership in Applied Health Research and Care WessexUniversity of SouthamptonSouthamptonHamphsireUK

**Keywords:** Sarcopenia, Very old, Risk factors, Prevalence, Incidence

## Abstract

**Introduction:**

Recognition that an older person has sarcopenia is important because this condition is linked to a range of adverse outcomes. Sarcopenia becomes increasingly common with age, and yet there are few data concerning its descriptive epidemiology in the very old (aged 85 years and above). Our aims were to describe risk factors for sarcopenia and estimate its prevalence and incidence in a British sample of the very old.

**Methods:**

We used data from two waves (2006/07 and 2009/10) of the Newcastle 85+ Study, a cohort born in 1921 and registered with a Newcastle/North Tyneside general practice. We assessed sarcopenia status using the European Working Group on Sarcopenia in Older People (EWGSOP) definition. Grip strength was measured using a Takei digital dynamometer (Takei Scientific Instruments Ltd., Niigata, Japan), gait speed was calculated from the Timed Up and Go test, and lean mass was estimated using a Tanita‐305 body fat analyzer. We used logistic regression to examine associations between risk factors for prevalent sarcopenia at baseline and incident sarcopenia at follow‐up.

**Results:**

European Working Group on Sarcopenia in Older People sarcopenia was present in 21% of participants at baseline [149/719 participants, mean age 85.5 (0.4) years]. Many participants had either slow gait speed or weak grip strength (74.3%), and hence measurement of muscle mass was frequently indicated by the EWGSOP definition. Incidence data were available for 302 participants, and the incident rate was 3.7 cases per 100 person years at risk. Low Standardized Mini‐Mental State Examination, lower occupational social class, and shorter duration of education were associated with sarcopenia at baseline, while low muscle mass was associated with incident sarcopenia. Low body mass index (BMI) was a risk factor for both in a graded fashion, with each unit decrease associated with increased odds of prevalent [odds ratio (OR) 1.29, 95% confidence interval (CI): 1.21, 1.37] and incident (OR 1.20, 95% CI: 1.08, 1.33) sarcopenia.

**Conclusions:**

To our knowledge, this is the first study to describe prevalence and incidence of EWGSOP sarcopenia in the very old. Low BMI was a risk factor for both current and future sarcopenia; indeed, there was some evidence that low BMI may be a reasonable proxy for low lean mass. Overall, the high prevalence of sarcopenia among the very old suggests that this group should be a focus for future research.

## Introduction

Sarcopenia is the loss of muscle mass and function that occurs with ageing, and this condition has been associated with a range of adverse outcomes including disability.^1^ A commonly used approach to diagnosis is that proposed by the European Working Group on Sarcopenia in Older People (EWGSOP), where the presence of slow gait speed or weak grip strength is an indication to test for low muscle mass.^2^ Those aged 80 years and above represent the fastest growing sector of the population worldwide^3^ and the focus of many clinicians who care for older people, and yet there are few data concerning the descriptive epidemiology of sarcopenia in the very old. For example, in a recent systematic review of 18 studies of sarcopenia prevalence,^4^ all but two had a mean age under 85 years. This may reflect the challenges of studying sarcopenia in the very old, such as the time required for assessment and factors such as medical illness that may make it difficult to obtain a representative sample.^5^ Estimates of prevalence are useful for the planning of services and clinical research. Such studies can also be used to identify risk factors for sarcopenia and hence which groups may stand to benefit most from assessment and intervention. There is also little published research on the incidence of sarcopenia, and existing studies have typically been conducted at mean ages below 75.^6^, ^7^ Our aims were therefore to estimate the prevalence and incidence of sarcopenia and to examine risk factors for both using a British sample of the very old.

## Methods

### Participants

We used data from two waves (2006/07, Wave 1 and 2009/10, Wave 3) of the Newcastle 85+ Study. Full details of the study have been published previously^8^, ^9^; in brief, the cohort were born in 1921 and were recruited to the study through Newcastle/North Tyneside general practices at around age 85. Of the 1040 people recruited to the study, 845 completed a multidimensional health assessment by a trained research nurse at their usual place of residence (whether this was at home or in an institution) and had a review of their general practice records. Ethical approval was obtained from Newcastle and North Tyneside Local Research Ethics Committee One, and informed written consent was obtained from all participants.

### Assessment of sarcopenia status

We assessed sarcopenia status at baseline and 3 year follow‐up using the EWGSOP definition^2^: slow gait speed or weak grip strength are indications to test for low muscle mass, which if present confirms the diagnosis of sarcopenia. Gait speed was not measured directly in Newcastle 85+ Study, although the Timed Up and Go (TUG) test was included: a stopwatch was used to measure the time taken to get up from a chair and walk as quickly and safely as possible up to and around a marker placed 3 m away, walk back to the chair, and sit back down. We converted this time to an estimate of gait speed using the formula [6/(TUG time)]*1.62.^10^, ^11^ We used the standard cut‐off of ≤0.8 m/s for slow gait speed.^2^

Hand grip strength (kg) was measured using a Takei A5401 digital dynamometer (Takei Scientific Instruments Ltd., Niigata, Japan) in the standing position, with two trials from each hand alternately. We used the maximum of the available measures for analyses. We used the cut‐offs for weak grip strength derived by the Foundation for the National Institute of Health (FNIH) sarcopenia project, <16 kg in women and <26 kg in men.^12^

We assessed muscle mass using bioimpedance values measured with a Tanita‐305 body fat analyzer (Tanita Corp., Tokyo, Japan). Participants were measured standing with bare feet placed on the metal sole plates of the device.^13^ We used the formula developed by Jansen *et al.* 14 to estimate skeletal muscle index (SMI, skeletal muscle mass divided by height squared, kg/m^2^) values in the participants. We used previously published cut‐offs for low SMI of <8.87 kg/m^2^ and <6.67 kg/m^2^ in men and women, respectively.^2^, ^15^

### Potential risk factors for sarcopenia

We identified potential risk factors for prevalent and incident sarcopenia *a priori* at wave 1 that we considered to be clinically relevant. These included difficulty with activities of daily living (ADLs, both basic and instrumental^16^), living in sheltered or institutional accommodation, the number of longstanding illnesses^17^ and prescribed medications, Standardized Mini‐Mental State Examination (SMMSE) score, Geriatric Depression Scale (GDS), body mass index (BMI), smoking history, self‐reported physical activity,^18^ occupational social class (or their partner's, where they were the main earner), and years of education. We also examined if pre‐sarcopenia (low muscle mass with normal gait speed and grip strength^2^) was a risk factor for incident sarcopenia.

### Statistical analyses

We considered that those unable to complete the TUG or grip strength measures due to health problems had low performance for the purpose of analyses. We used univariable logistic regression to examine associations between each risk factor and prevalent sarcopenia at baseline and incident sarcopenia at follow‐up. We set our level for statistical significance as *P* < 0.05. We considered it likely that many of those who died before follow‐up had developed sarcopenia prior to their death. We therefore repeated our incidence analyses using a combined end‐point of sarcopenia and death. We performed all analyses using Stata version 14.0.^19^

## Results

We were able to assign a status of normal or low to the gait speed, grip strength, and muscle mass of 719 participants at baseline (85% of those who underwent health assessment and GP record review), including those who were unable to complete the TUG (*n* = 4) or grip strength (*n* = 2) tests due to health reasons. The characteristics of those included in the study are shown in *Table* 1. The majority were women (61%), had difficulty with between one and five ADLs, and had three or more chronic diseases. Mean BMI was 24.4 (4.4) kg/m^2^.

**Table 1:** Distribution of risk factors by sarcopenia status

Risk factor	All		No sarcopenia		Sarcopenia	
(*n* = 719 unless shown otherwise)	No.	(%)	No.	(%)	No.	(%)
Gender						
Male	282	(39.2)	223	(39.1)	59	(39.6)
Female	437	(60.8)	347	(60.9)	90	(60.4)
ADLs with difficulty (*n* = 713)						
None	156	(21.9)	131	(23.1)	25	(17.1)
1–5	352	(49.4)	280	(49.4)	72	(49.3)
6 or more	205	(28.8)	156	(27.5)	49	(33.6)
Type of housing						
Standard	593	(82.5)	474	(83.2)	119	(79.9)
Sheltered	119	(16.6)	90	(15.8)	29	(19.5)
Institution	7	(1.0)	6	(1.1)	1	(0.7)
Disease count						
0 or 1	212	(29.5)	168	(29.5)	44	(29.5)
2	219	(30.5)	175	(30.7)	44	(29.5)
3 or more	288	(40.1)	227	(39.8)	61	(40.9)
Total prescribed medications						
0–4	242	(33.7)	192	(33.7)	50	(33.6)
5–7	239	(33.2)	194	(34.0)	45	(30.2)
8 or more	238	(33.1)	184	(32.3)	54	(36.2)
SMMSE (*n* = 718)						
26–30 (normal)	545	(75.9)	444	(78.0)	101	(67.8)
21–25 (mild impairment)	124	(17.3)	90	(15.8)	34	(22.8)
0–20 (severe impairment)	49	(6.8)	35	(6.2)	14	(9.4)
GDS (if SMMSE ≥15) (*n* = 716)						
0–5 (no depression)	563	(78.6)	453	(79.8)	110	(74.3)
6–7 (mild depression)	85	(11.9)	64	(11.3)	21	(14.2)
8 or more (severe depression)	52	(7.3)	40	(7.0)	12	(8.1)
(SMMSE <15)	16	(2.2)	11	(1.9)	5	(3.4)
BMI (kg/m^2^) (*n* = 716)						
Under 18.5	46	(6.4)	19	(3.4)	27	(18.1)
18.5–24.9	369	(51.5)	271	(47.8)	98	(65.8)
25 or above	301	(42.0)	277	(48.9)	24	(16.1)
Smoking status (*n* = 718)						
Never	245	(34.1)	193	(33.9)	52	(35.1)
Current	42	(5.8)	29	(5.1)	13	(8.8)
Former	431	(60.0)	348	(61.1)	83	(56.1)
Physical activity (*n* = 717)						
Low	132	(18.4)	94	(16.5)	38	(25.5)
Medium	319	(44.5)	256	(45.1)	63	(42.3)
High	266	(37.1)	218	(38.4)	48	(32.2)
Own/partner's occupation (*n* = 694)						
Managerial/professional	241	(34.7)	203	(36.9)	38	(26.4)
Intermediate	83	(12.0)	66	(12.0)	17	(11.8)
Routine/manual	370	(53.3)	281	(51.1)	89	(61.8)
Years in education (*n* = 715)						
0–9	459	(64.2)	359	(63.3)	100	(67.6)
10–11	166	(23.2)	129	(22.8)	37	(25.0)
12–20	90	(12.6)	79	(13.9)	11	(7.4)

ADLs, activities of daily living; BMI, body mass index; GDS, Geriatric Depression Scale; SMMSE, Standardized Mine‐Mental State Examination.

### Prevalence and incidence of sarcopenia

**Figure 1:**
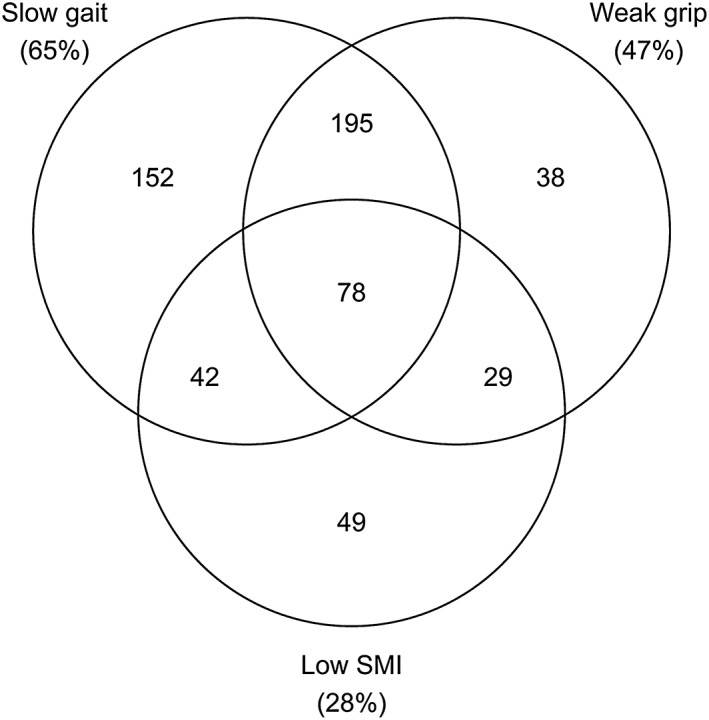
Overlap between sarcopenia components at baseline. This figure shows the number of participants from the baseline assessment (total *n*=719) with each combination of slow gait, weak grip, and low skeletal muscle index (SMI). The percentage of the total sample with a low value for each of the three measures is shown in brackets.

The overall prevalence of sarcopenia at baseline was 21% at mean age 85.5 (0.4) years, with similar findings in men and women. Mean estimated gait speed (m/s) was 0.8 (0.3) and 0.7 (0.3) in men and women, respectively, whereas mean grip strength (kg) was 27.3 (7.1) and 15.3 (4.8). These mean values were similar to the sarcopenia cut‐points used, with 74% of participants falling below one or both and therefore requiring assessment of muscle mass by the EWGSOP definition (*Figure* 1). Mean estimated SMI (kg/m^2^) was 9.9 (1.8) in men and 7.7 (2.2) in women, with 28% of participants falling below the cut‐points used.

**Figure 2:**
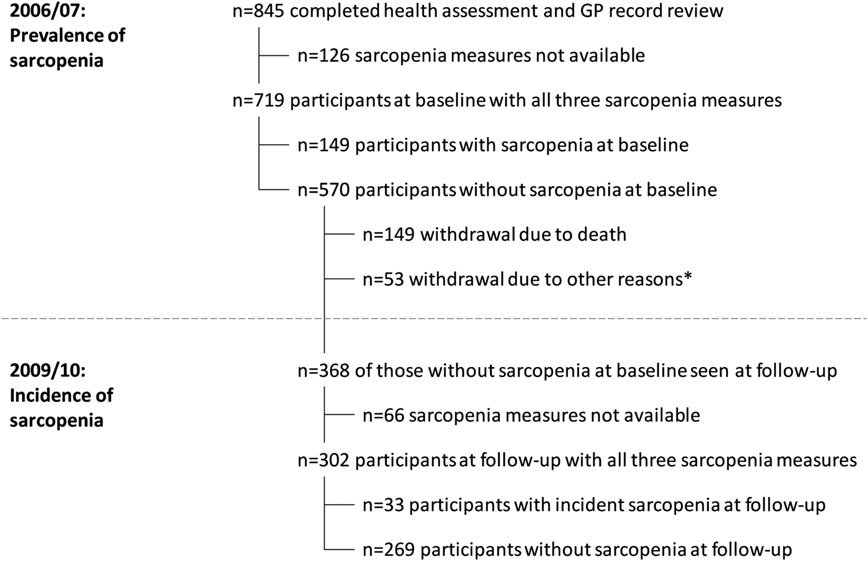
Flow diagram of participants. Only potential incident cases are shown at follow‐up, that is, those participants without sarcopenia at baseline. ‘Asterisks’, other reasons for withdrawal included ill health, fatigue, and losing interest in the study.

Follow‐up data were available for 302 of the 570 participants at baseline without sarcopenia. There were 149 withdrawals from the study because of death as shown in *Figure* 2. The mean follow‐up time was 2.99 (0.04) years, and there were 33 incident cases of sarcopenia: an incidence rate of 3.66 cases per 100 person years at risk.

### Risk factors for sarcopenia

The associations of the potential risk factors with prevalent and incident sarcopenia are shown in *Table* 2. We saw that those with evidence of cognitive impairment (MMSE <26) were at increased risk of prevalent sarcopenia (OR 1.64 (95% CI: 1.07, 2.52), *P* = 0.03), while those with normal or raised BMI were at reduced risk. Participants with BMI 25 or above had 14 times lower odds [OR 0.07 (95% CI: 0.03, 0.15), *P* < 0.01] than those with BMI <18.5. There was also evidence that those whose occupation (or partner's occupation) was routine/manual were at increased risk, as were those with fewer than 12 years in education.

**Table 2:** Association between risk factors and prevalent/incident sarcopenia

	Association between risk factor and outcome shown					
	Prevalent sarcopenia (*n* = 664)^a^			Incident sarcopenia (*n* = 290)^b^		
Risk factor	OR	(95% CI)	*P*	OR	(95% CI)	*P*
Gender			0.77			0.59
Male	1			1		
Female	1.06	(0.72, 1.56)		1.23	(0.58, 2.60)	
ADLs with difficulty			0.37			0.04
None	1			1		
1–5	1.37	(0.83, 2.28)		1.55	(0.69, 3.52)	
6 or more	1.45	(0.83, 2.56)		0.23	(0.03, 1.86)	
Type of housing^c^			0.39			0.36
Standard	1			1		
Sheltered	1.26	(0.75, 2.10)		1.66	(0.59, 4.68)	
Disease count			0.99			0.67
0 or 1	1			1		
2	0.96	(0.59, 1.57)		1.01	(0.43, 2.38)	
3 or more	0.98	(0.62, 1.55)		0.7	(0.28, 1.75)	
Total prescribed medications			0.83			0.83
0–4	1			1		
5–7	0.88	(0.55, 1.40)		0.78	(0.33, 1.82)	
8 or more	1	(0.63, 1.58)		0.83	(0.33, 2.07)	
SMMSE^c^			0.03			0.16
26–30 (normal)	1			1		
15–25	1.64	(1.07, 2.52)		0.4	(0.09, 1.73)	
GDS^c^			0.61			0.09
0–5 (no depression)	1			1		
6–7 (mild depression)	1.31	(0.75, 2.29)		3.27	(1.18, 9.10)	
8 or more (severe depression)	1.17	(0.58, 2.36)		0.62	(0.08, 4.94)	
BMI (kg/m^2^)			<0.01			<0.01
Under 18.5	1			1		
18.5–24.9	0.29	(0.15, 0.56)		0.77	(0.20, 2.95)	
25 or above	0.07	(0.03, 0.15)		0.17	(0.04, 0.77)	
Smoking status			0.13			0.22
Never	1			1		
Current	1.99	(0.95, 4.18)		2.89	(0.52, 16.04)	
Former	0.93	(0.62, 1.41)		1.99	(0.82, 4.79)	
Physical activity^c^			0.31			0.55
Low/medium	1			1		
High	0.82	(0.55, 1.21)		1.25	(0.60, 2.58)	
Own/partner's occupation			0.03			0.16
Managerial/professional	1			1		
Intermediate	1.3	(0.67, 2.53)		1.26	(0.37, 4.34)	
Routine/manual	1.76	(1.14, 2.73)		2.16	(0.94, 4.96)	
Years in education			0.08			0.59
0–9	1			1		
10–11	0.85	(0.53, 1.34)		0.85	(0.34, 2.09)	
12–20	0.48	(0.24, 0.96)		0.58	(0.19, 1.75)	
Pre‐sarcopenia (incidence only)						<0.01
SMI above cut‐points				1		
SMI below cut‐points				8.28	(3.64, 18.84)	

ADLs, activities of daily living; BMI, body mass index; GDS, Geriatric Depression Scale; SMMSE, Standardized Mine‐Mental State Examination.

a *n* = 664 (of 719) in the prevalence sample with complete information on risk factors shown.

b *n* = 290 (of 302) in the incidence sample with complete information on risk factors shown.

c The smaller sample for the incidence analyses meant levels of some variables had too few participants for inclusion or predicted the outcome exactly. We therefore removed those unable to complete the GDS as their MMSE was below 15 and those living in institutions. These changes excluded 14 participants from the prevalence analyses. We also grouped those remaining with MMSE <26 together and those with low/medium physical activity together.

In terms of incident sarcopenia, higher BMI again appeared protective with an OR of 0.17 (95% CI: 0.04, 0.77), *P* < 0.01. The presence of low SMI, or pre‐sarcopenia, at baseline was also strongly associated with incident sarcopenia (OR 8.28 (95% CI: 3.64, 18.84), *P* < 0.01). We repeated our analyses using a combined outcome of incident sarcopenia or death. As shown in Appendix 1, we found strong positive relationships with multiple risk factors: ADL disability, living in sheltered housing, number of chronic diseases, number of prescription medications, lower MMSE, higher GDS, low/medium physical activity, lower occupational class, and shorter duration of education. We no longer saw the relationships with low BMI and low SMI when death was included as an alternative outcome alongside sarcopenia.

## Discussion

### Summary of findings

To our knowledge, this is the first study to describe prevalence and incidence of EWGSOP‐defined sarcopenia in those aged 85 years and over. Sarcopenia was present in 21% of the participants at baseline. Of those without sarcopenia at baseline, approximately 10% developed sarcopenia at follow‐up after an average of 3 years. Low BMI predicted both prevalent and incident sarcopenia, whereas other risk factors such as number of chronic diseases showed less evidence of an association. There was also substantial loss to follow‐up, mainly due to death. When we included death as an alternative outcome alongside incident sarcopenia, we saw much stronger relationships with the risk factors examined.

### Interpretation of findings

The Newcastle 85+ Study was broadly representative at baseline of those born in 1921 and registered with a Newcastle upon Tyne or North Tyneside general practice.^9^ We included the majority of the baseline sample, although there were exclusions, particularly among those living in institutions; this group made up 1% of our sample at baseline, compared with 5% of those who took part in the study. As such, it seems likely that the true prevalence of sarcopenia may be higher than 21%.

We are aware of four other studies that have examined the prevalence of EWGSOP sarcopenia at mean age 85 years or above. Their findings varied, but in all four there was a high proportion of individuals (81% or greater) who fell below gait speed and grip strength cut‐points and therefore required assessment of muscle mass. Legrand *et al.* 20 found a prevalence of 12.5% in a Belgian sample at mean age 84.8 (3.6) years using bioelectrical impedance analysis (BIA) to assess muscle mass. Landi *et al.* 21 and Senior *et al.* 22 also used BIA and found prevalences of 32.8% and 40.2% among residents in nursing homes at mean ages 84.1 (6.9) and 84.5 (8.2) years, respectively. Finally, Landi *et al.* 23 used mid‐arm muscle circumference to estimate muscle mass and found a prevalence of 29.1% in an Italian sample at mean age 85.8 (4.9) years.

We are not aware of other studies that have examined the incidence of EWGSOP sarcopenia in this age group. Of those without sarcopenia at baseline who completed follow‐up, we saw an average annual incidence of 3.6%. This is similar to the annual incidence of 3.4% found by Yu *et al.* in a Chinese sample aged 72.5 (5.2) years at baseline.^6^ There were also approximately four times as many participants lost to follow‐up because of death as there were incident cases of sarcopenia. It is likely that a substantial proportion of those who died had developed sarcopenia prior to their death^24^, and hence our incidence rate may also be an underestimate.

In terms of risk factors for sarcopenia, we saw evidence of cross‐sectional associations with low SMMSE, low socio‐economic position, and especially with low BMI. This mirrors other findings in this age group^21^, ^22^ in which an association with higher levels of ADL disability has also been found.^20^ In longitudinal analyses in our study (*n* = 290), low BMI and low SMI were clearly associated with sarcopenia. In the study by Yu *et al.* (*n* = 2898 for 4 year follow‐up of incident sarcopenia), associations were also found with impairments of instrumental ADLs, lower physical activity, and the presence of chronic obstructive pulmonary disease and stroke.^6^ It may be that there are also associations with these factors in our sample population, but that our study is inadequately powered to detect them.

The strong relationships between BMI and sarcopenia, both prevalent and incident, deserve further consideration. These appear to have arisen in our sample because the presence or absence of sarcopenia was largely dependent on SMI status (as described earlier). In turn, BMI is positively correlated with SMI, with low SMI being uncommon at BMI 25 and above. Indeed, it has previously been suggested that skeletal muscle mass estimated from bioimpedance provides little additional information regarding body composition than that from BMI.^25^ In additional analyses (not shown), we included both low SMI and BMI in the same model, and both remained significantly associated with incident sarcopenia. This would suggests that SMI estimated from bioimpedance provides useful information on muscle mass in addition to that from BMI, and that both are risk factors for the development of sarcopenia.

### Strengths and limitations

This study may have been underpowered to detect associations with potential risk factors, particularly for incident sarcopenia. We also used BIA when dual energy X‐ray absorptiometry (DEXA) is the preferred method for assessment of muscle mass.^2^ However, BIA has been used in other studies and has the advantage that the equipment is portable. Strengths of this study include the extensive efforts during the Newcastle 85+ Study fieldwork to recruit a representative sample of general practices and participants and to record reasons for non‐participation with measures such as grip strength. We also included withdrawal due to death as a competing risk in our analyses on incident sarcopenia.

### Implications for clinical practice and future research

This study adds to the existing literature which suggests that the assessment of EWGSOP sarcopenia is feasible in the very old, at least in a research setting. The high prevalence of individuals requiring assessment of muscle mass is a challenge, particularly if the hospital‐based technique of DEXA is to be used.^26^ There was some suggestion from our results that BMI could be used as an initial screen for those likely to have low skeletal muscle mass before undertaking formal testing. There is also interest in using questionnaire‐based measures such as the SARC‐F tool to screen those who require further assessment of possible sarcopenia.^27^

We found that several common clinical factors such as number of prescribed medications were not associated with prevalent and/or incident sarcopenia. This may be because our study lacked sufficient power to detect such associations. By contrast, when we included study withdrawal due to death as an alternative outcome alongside incident sarcopenia, we saw clear associations with many of the risk factors tested (Appendix 1). This highlights a challenge of exploring risk factors for incidence in this age group, where surviving to follow‐up and undergoing assessment of disease status is likely to be a marker of better overall health.

The Foundation for the National Institutes of Health Biomarkers Consortium have developed cut‐points for grip strength and for appendicular lean mass divided by body mass index (ALM_BMI_).^28^ They undertook validation of the cut‐points in terms of mortality and incident disability and recommended that further validation studies for each measure be carried out.^29^ The findings from the present study support this approach and also highlight the need to conduct analyses that incorporate competing risks (such as mortality) in the very old.

## Conclusions

We investigated the prevalence and incidence over 3 years of sarcopenia using the EWGSOP definition in a representative sample of the very old, the Newcastle 85+ Study. There was a high prevalence of sarcopenia and a high incidence of sarcopenia or death during follow‐up. We explored a range of risk factors and saw the strongest relationships with low BMI. This is of potential clinical relevance, especially when measurement of muscle mass may not be feasible. The high prevalence of sarcopenia among the very old suggests that this group should be a particular focus for future research.

## Acknowledgements

R. D. is funded by the NIHR Integrated Academic Training Programme.

A. A. S. is Director of the NIHR Newcastle Biomedical Research Centre in Ageing and Chronic Disease.

A. G. is supported by the NIHR Newcastle Biomedical Research Centre in Ageing and Chronic Disease based at Newcastle Hospital Foundation Trust and Newcastle University.

The Newcastle 85+ Study was supported by the UK Medical Research Council and Biotechnology and Biological Sciences Research Council (G0500997, G0601333) and by the Dunhill Medical Trust.

Ethical approval for this study was obtained from Newcastle and North Tyneside Local Research Ethics Committee One, and informed written consent was obtained from all participants.

The authors certify that they comply with the ethical guidelines for publishing in the Journal of Cachexia, Sarcopenia and Muscle: update 2015.^30^

## Conflicts of interest

Richard Dodds, Antoneta Granic, Karen Davies, Thomas Kirkwood, Carol Jagger, and Avan Sayer declare that they have no conflict of interest.

## Appendix 1

Association between risk factors and combined outcome of incident sarcopenia or death

**Table:**

	Association between factor shown and incident sarcopenia or death (*n* = 424)^a^		
Risk factor	OR	(95% CI)	*P*
Gender			0.32
Male	1	(1.00, 1.00)	
Female	0.82	(0.55, 1.22)	
ADLs with difficulty			<0.01
None	1	(1.00, 1.00)	
1–5	2.22	(1.30, 3.79)	
6 or more	5.35	(2.90, 9.86)	
Type of housing			<0.01
Standard	1		
Sheltered	2.29	(1.30, 4.01)	
Disease count			0.01
0 or 1	1		
2	1.01	(0.60, 1.71)	
3 or more	1.84	(1.15, 2.97)	
Total prescribed medications			<0.01
0–4	1		
5–7	1.4	(0.86, 2.29)	
8 or more	2.36	(1.45, 3.84)	
SMMSE			0.01
26–30 (normal)	1		
25 or below	1.93	(1.17, 3.19)	
GDS (if MMSE ≥15)			<0.01
0–5 (no depression)	1		
6–7 (mild depression)	2.93	(1.51, 5.66)	
8 or more (severe depression)	1.93	(0.90, 4.13)	
BMI (kg/m^2^)			0.22
Under 18.5	1		
18.5–24.9	1.22	(0.45, 3.26)	
25 or above	0.85	(0.32, 2.29)	
Smoking status			0.03
Never	1		
Current	2.53	(0.95, 6.70)	
Former	1.7	(1.09, 2.64)	
Physical activity			<0.01
Low/medium	1		
High	0.5	(0.33, 0.75)	
Own/partner's occupation			0.06
Managerial/professional	1		
Intermediate	0.96	(0.51, 1.83)	
Routine/manual	1.6	(1.05, 2.44)	
Years in education			0.02
0–9	1		
10–11	0.92	(0.57, 1.48)	
12–20	0.42	(0.22, 0.79)	
Pre‐sarcopenia			0.2
SMI above cut‐points	1		
SMI below cut‐points	1.53	(0.80, 2.92)	

ADLs, activities of daily living; BMI, body mass index; GDS, Geriatric Depression Scale; SMMSE, Standardized Mine‐Mental State Examination.

a *n* = 424 (of 451) in the sample with follow‐up for incident sarcopenia (or death before follow‐up) and complete information on risk factors shown.

We also repeated the aforementioned analyses with deaths within 6 months of the baseline assessment excluded (*n* = 14) to see if any of the aforementioned associations were reflective of pre‐terminal illness. The results were unchanged when we did this.
